# Teenagers in Face of the War in Ukraine: Experience of Lithuanians

**DOI:** 10.15388/Amed.2026.33.1.9

**Published:** 2026-07-22

**Authors:** Sigita Lesinskienė, Augustė Lapinskaitė, Augustė Česnauskaitė

**Affiliations:** 1 Clinic of Psychiatry, Institute of Clinical Medicine, Faculty of Medicine, Vilnius University, Vilnius, Lithuania E-mail: ORCID iD; 2 Faculty of Medicine, Vilnius University, Vilnius, Lithuania E-mail:; 3 Lithuanian University of Health Sciences, Kaunas, Lithuania E-mail:

**Keywords:** adolescents, teenagers, war, mental health, Ukraine, Lithuania, Raktiniai žodžiai: paaugliai, karas, emocinė sveikata, Ukraina, Lietuva

## Abstract

**Background:**

The war in Ukraine, starting on February 24, 2022, shocked Lithuanians who actively support Ukraine and who are still experiencing the threat of an extended war. Adolescents are a sensitive group in society. They go through many changes at their age and are in the process of revealing their feelings and forming their worldviews. The study aimed to investigate how war events in Ukraine affected Lithuanian adolescents’ everyday life, relationships, health, attitudes, concentration, and learning.

**Materials and methods:**

Adolescents in 6 high schools filled out an anonymous *Google Forms* survey developed by the authors comprising general data about the respondent, their interest in war events and their involvement in following the news about the war in media, the impact of war events on their physical and mental health, and the feeling of fear about the threats of the war in Lithuania.

**Results:**

395 adolescents aged 16–19 (mean age 17.01±0.97) filled in the questionnaire. This research revealed that adolescents in Lithuania are moderately interested in war events in Ukraine, and 25.3% noted that they follow news about the war once a week. Most teenagers did not note that the ongoing war in Ukraine would affect their communication, ability to concentrate and learn, hobbies, appetite, and sleep. However, 48.9% indicated that war events affected their emotions and mood. Adolescents who talked about the ongoing war with their parents (55.4%, *p*=0.00006), who were more interested in war events in the media (median=229.7, *p*<0.001), and girls (53.3%, *p*=0.008) were statistically significantly more likely to notice that the ongoing war would affect their emotions and mood. 60.8% of the respondents felt fear and threat that war may break out in Lithuania as well.

**Conclusions:**

The news in the media and the constant threat of war affect Lithuanian youth, mostly their emotions and mood. Therefore, it is crucial to research and monitor their emotional health, discuss things with teenagers, and provide them with a possibility to share their views, thoughts, perceptions, fears, and feelings. Talking with youth about the war situation responsibly, promptly, and age appropriately is essential.

## Abstract (it)

**Įvadas:**

2022 m. vasario 24 d. Ukrainoje prasidėjęs karas sukrėtė lietuvius, jie aktyviai remia Ukrainą ir vis dar jaučia tebesitęsiančio karo grėsmę. Paaugliai yra pažeidžiama visuomenės grupė. Šiuo amžiaus tarpsniu jie išgyvena intensyvius pokyčius, mokosi atpažinti ir reikšti savo jausmus, formuojasi jų pasaulėžiūra. Šio tyrimo tikslas buvo apklausti ir išsiaiškinti, kaip Ukrainoje vykstantis karas paveikė Lietuvos paauglių kasdienį gyvenimą, santykius, emocinę ir fizinę sveikatą, gebėjimą susikaupti ir mokytis.

**Metodika:**

Šešiose gimnazijose paaugliai pildė autorių sukurtą anoniminę *Google forms* anketą. Anketos pradžioje buvo pateikti demografiniai duomenys apie respondentą, buvo klausiama apie domėjimąsi karo įvykiais ir naujienų stebėjimą žiniasklaidoje, karo įvykių poveikį jų fizinei ir psichinei sveikatai, baimės dėl karo grėsmės Lietuvoje jausmą.

**Rezultatai:**

Anketą užpildė 395 16–19 metų (amžiaus vidurkis 17,01±0,97) paaugliai. Šis tyrimas atskleidė, kad Lietuvos paaugliai vidutiniškai domisi karo įvykiais Ukrainoje, o 25,3 % pažymėjo, kad naujienas apie karą seka kartą per savaitę. Dauguma paauglių nenurodė, kad Ukrainoje vykstantis karas paveikė jų bendravimą, gebėjimą susikaupti ir mokytis, pomėgius, apetitą ir miegą. Tačiau 48,9 % respondentų teigimu, kad įvykiai paveikė jų emocijas ir nuotaiką. Paaugliai, kurie kalbėjosi apie vykstantį karą su tėvais (55,4 %, p = 0,00006), kurie labiau domėjosi karo įvykiais žiniasklaidoje (mediana = 229,7, p < 0,001), ir merginos (53,3 %, p = 0,008), statistiškai reikšmingai dažniau teigė, kad vykstantis karas paveikė jų emocijas ir nuotaiką. 60,8 % respondentų jaučia baimę ir grėsmę, kad karas gali prasidėti ir Lietuvoje.

**Išvados:**

Žiniasklaidoje skelbiamos naujienos ir nuolatinė karo grėsmė turi poveikį Lietuvos jaunimui, labiausiai jų emocijoms ir nuotaikai. Todėl labai svarbu tirti ir stebėti jų emocinę sveikatą, diskutuoti su paaugliais ir suteikti galimybę dalytis savo požiūriu, mintimis, suvokimu, baimėmis ir jausmais. Labai svarbu atsakingai, laiku ir amžiui tinkamais būdais kalbėtis su paaugliais apie karo situaciją.

## Introduction

Adolescence is a critical period of emotional and psychological development, when young people navigate personal identity formation, increasing independence, and growing awareness of global issues [1,2]. Exposure to major world events, such as war and geopolitical conflicts, can significantly shape their perspectives, emotional well-being, and sense of security [3,4]. The ongoing war in Ukraine, which began on February 24, 2022, has not only reshaped political landscapes but has also profoundly impacted societies beyond the immediate warzone [2,4,5]. Lithuania, as a neighbouring country with historical ties to Ukraine and a shared history of Russian aggression, has been particularly affected. The Lithuanian Government and society have actively supported Ukraine, while simultaneously facing heightened concerns about national security [6]. For adolescents, this situation presents a unique psychological challenge – that of balancing the awareness of war with their own sense of safety and stability. Given Lithuania’s geographical proximity to Ukraine and its deep-rooted historical experiences with occupation and conflict, it is crucial to examine how Lithuanian youth perceive and emotionally process war events. While previous studies have examined how direct exposure to war impacts children and adolescents in conflict zones, less attention has been given to how youth in neighbouring countries, in which, direct military action is not taking place, are affected by media exposure and the perceived threat of war. Psychological research has shown that global crises can heighten anxiety and uncertainty among adolescents, particularly when they involve themes of violence, displacement, and instability [8]. However, the extent to which war events in Ukraine influence the daily lives, emotions, and mental health of Lithuanian teenagers remains an important area of investigation. This study intends to explore how the war in Ukraine has affected Lithuanian adolescents, while focusing on their everyday lives, relationships, emotional well-being, and sense of security. The research examines their interest in following war-related news, discussions with parents and peers, and whether the war has influenced their ability to concentrate, learn, sleep, or engage in hobbies. By understanding their perspectives, we can gain valuable insight into the broader psychological effects of war on adolescents who, while not being directly affected, still experience its impact through media exposure and social conversations. To achieve this objective, an anonymous survey was conducted among adolescents aged 16–19 in six high schools in Lithuania. The questionnaire gathered data on general demographics, interest in war-related news, and the perceived effects of war on various aspects of their well-being. The study also sought to identify potential gender differences and whether discussions with parents or higher media exposure levels influence emotional responses. By analyzing these factors, we aim to provide a clearer picture of how young people in Lithuania are processing and coping with the ongoing conflict. Understanding the emotional responses of adolescents to war events is crucial for shaping educational approaches, mental health strategies, and social support systems. If war-related anxiety and emotional distress are significant among Lithuanian youth, steps must be taken to address their concerns in schools, families, and public discourse. By fostering open conversations, promoting media literacy, and providing psychological support, we can help teenagers navigate these challenging times with greater resilience.

## Materials and Methods

### Participants and procedure

The study was implemented in 2023, from March to April, and in 2024, from January to March. Six high schools from four Lithuanian cities (Vilnius, Kaunas, Klaipėda and Tauragė) participated in the survey, with the permission of their administrative bodies. A total of 395 adolescents aged 16–19 from six high schools completed the anonymous survey. All study participants were adolescents who voluntarily agreed to complete the questionnaire. A description of the study was provided at the beginning of the questionnaire, in which, the respondents were informed about the purpose of the study and were made aware of their right to refuse. The respondents were also informed that the information obtained from them was anonymous and secure, and would be treated with complete confidentiality. The only criterion for inclusion was that the age of the participants had to be from 16 to 19 years.

### Instruments

An anonymous *Google Forms* questionnaire compiled by the authors was used in the course of the study. It was written in the Lithuanian language and had 14 questions. The questionnaire consisted of questions about the respondent’s demographic data (age, gender, city where they attend school), the extent to which they are interested in the war in Ukraine, the frequency with which they follow news about the war in the media and on social networks, whether they discuss the events of the war with people in their close environment (friends, parents, teachers), how the war in Ukraine has affected the respondent’s emotional well-being and life, whether the ongoing war has affected communication with family members, friends, their general physical and mental health, emotions and mood, ability to concentrate and learn, hobbies, appetite, sleep, whether the respondent was involved in providing help to Ukrainians in any way by volunteering, participating in charitable activities, whether they had to communicate with Ukrainians who fled during the war to Lithuania, whether they maintain constant contact and communication with them, whether they feel safe from the war, and whether they feel that the war could also start within the territory of Lithuania.

### Statistical analysis

The collected data were manually coded by using *Microsoft Excel 2016*. Statistical analysis was conducted by using *Microsoft Excel 2016* and *IBM SPSS Statistics 27.0*. The results are presented in numerical and percentage formats, with continuous variables expressed as means and standard deviations. To compare quantitative values between two independent samples, the non-parametric Mann-Whitney U test was applied. The Chi-square (χ^2^) test was used to evaluate associations between ordinal variables. In cases where data were presented in a 2x2 contingency table and the expected frequency of observations was below five, the Fisher’s exact test was additionally employed. A *p*-value of less than 0.05 (*p*<0.05) was considered statistically significant.

## Results

395 adolescents aged 16–19 (mean age 17.01±0.97) filled in the questionnaires, of whom, 66.1% (n=261) were girls, 32.4% (n=128) were boys, and 1.5% (n=6) were non-binary gender.

Lithuanian adolescents’ interest in the war events in Ukraine

**Figure 1:**
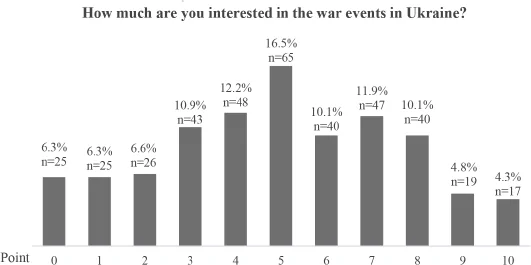
Lithuanian adolescents’ interest in the war events in Ukraine (0 points = not interested, 10 points = highly interested)

This research revealed that adolescents in Lithuania are moderately interested in the war events in Ukraine. Most of them (16.5%, n=65), in a Likert scale from ‘0’ to ‘10’, rated their interest in the war events by 5 points (Figure 1).

**Figure 2:**
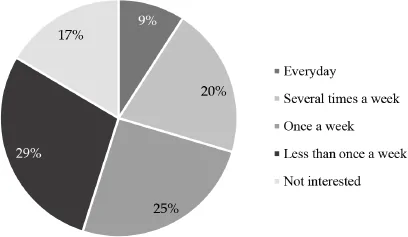
Frequency at which adolescents in Lithuania follow news about the war in Ukraine.

When asked how often they follow news in the media or on social networks about the war events in Ukraine, almost a third (28.6%, n=113) responded that they followed the news less than once a week, a quarter (25.3%, n=100) replied they followed it once a week, 20.5% (n=81) stated they follow it several times a week, whereas 16.5% (n=65) acknowledged that they did not follow the news at all, and only a small portion (9.1%, n=36) declared they followed it every day (Figure 2).

Talking about the war events in Ukraine with close people

When teenagers were asked if they discussed the events of the war in Ukraine with close people, the majority responded that they would talk with their parents (n=278, 70.4%) and with friends (n=229, 58.0%) (Table 1).

**Table 1:** Interlocutors of Lithuanian adolescents for talking about the war events in Ukraine

	Yes		No	
	*%*	*n*	*%*	*n*
**Parents**	58.0	229	42.0	166
**Friends**	70.4	278	29.6	117
**Relatives**	42.8	169	57.2	26
**Teachers**	35.4	140	64.6	255

Impact of the war in Ukraine on adolescents in Lithuania

**Figure 3:**
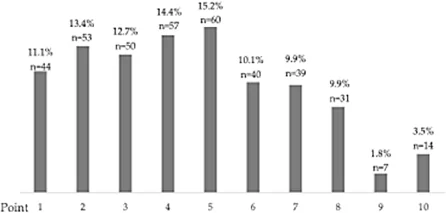
Impact of the war in Ukraine on the emotional well-being and everyday life of adolescents in Lithuania (1 point = not affected at all, 10 points = very strongly affected)

Out of the 395 adolescents surveyed in the study, the majority noted that the war in Ukraine had a moderate impact on their mental health and everyday life (Figure 3).

**Figure 4:**
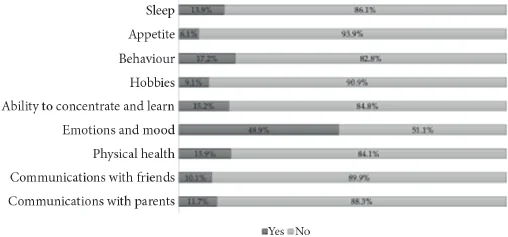
Adolescents’ opinion on whether the war in Ukraine has affected their specific habits

Most teenagers did not note that the ongoing war in Ukraine would somehow affect their communication, ability to concentrate and learn, hobbies, appetite, and sleep. However, 48.9% indicated that the war events affected their emotions and mood. Almost one fifth (17.2%, n=68) noted changes in their behaviour and 15.9% (n=63) observed changes in their physical health in the context of the ongoing war (Figure 4).

**Figure 5:**
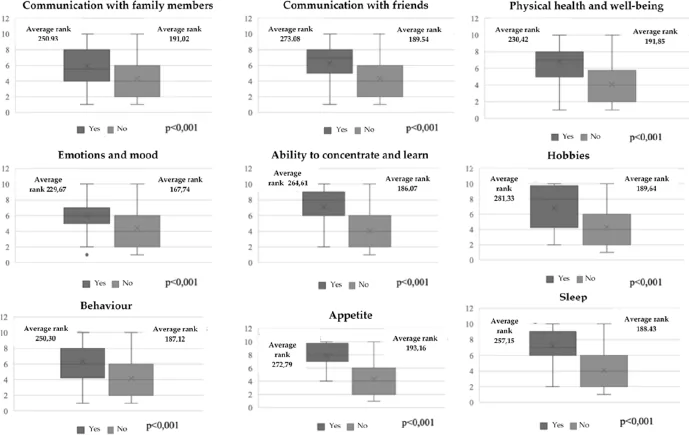
Relationship between adolescents’ interest in war events on the Likert scale and changes in habits

It was observed that those adolescents who gave higher scores on the Likert scale regarding their interest in the war events in Ukraine were statistically significantly more likely to indicate that the ongoing war had affected their communication with family members and friends, general physical health and well-being, emotions and mood, ability to concentrate and learn, hobbies, behaviour, appetite, and sleep (Figure 5).

When comparing the impact of the war in Ukraine on girls and boys, it was observed that girls were statistically significantly more likely to notice that the ongoing war affected their physical health and well-being (n=51, 19.5%), (*p*=0.001), emotions and mood (n=139, 51.5%), (*p*=0.008), appetite (n=22, 8.4%), (*p*=0.002), and sleep (n=47, 18.0%), (*p*=0.0008) (Table 2).

**Table 2:** Comparison between girls and boys on how the war in Ukraine affected their habits

			Communication with family members				Communication with friends				Physical health and well-being			
	Total		Yes		No		Yes		No		Yes		No	
	*%*	*n*	*%*	*n*	*%*	*n*	*%*	*n*	*%*	*n*	*%*	*n*	*%*	*n*
**Girls**	67.1	261	11.5	30	88.5	231	9.2	24	89.1	237	19.5	51	80.5	210
**Boys**	32.9	128	11.7	15	88.3	113	10.9	14	90.8	114	7.0	9	93.0	119
			*p=0.948*				*p=0.587*				*p= 0.001*			
			**Emotions and mood**				**Ability to concentrate and learn**				**Hobbies**			
	Total		Yes		No		Yes		No		Yes		No	
	*%*	*n*	*%*	*n*	*%*	*n*	*%*	*n*	*%*	*n*	*%*	*n*	*%*	*n*
**Girls**	67.1	261	53.3	139	46.7	122	17.6	46	82.4	215	8.8	23	91.2	238
**Boys**	32.9	128	39.1	50	60.9	78	10.2	13	89.8	115	9.4	12	90.6	116
			*p=0.008*				*p=0.054*				*p=0.855*			
			**Behaviour**				**Appetite**				**Sleep**			
	Total		Yes		No		Yes		No		Yes		No	
	*%*	*n*	*%*	*n*	*%*	*n*	*%*	*n*	*%*	*n*	*%*	*n*	*%*	n
**Girls**	67.1	261	18.0	47	82.0	214	8.4	22	91.6	239	18.0	47	82.0	214
**Boys**	32.9	128	14.8	19	85.2	109	0.8	1	99.2	127	5.5	7	94.5	121
			*p=0.435*				*p=0.002*				*p=0.0008*			

Adolescents who reported talking to their parents about the events of the war in Ukraine noted that the ongoing war had affected their overall physical health and well-being (n=53, 19.1%) (*p*=0.009) as well as emotions and mood (n=154, 55.4%) (*p*=0.00006) compared to those who reported not talking to their parents. Also, these changes were found to be statistically significant (Table 3).

**Table 3:** Impact of the war in Ukraine has affected on habits of adolescents who talk and who do not talk to their parents about the war

			Communication with family members				Communication with friends				Physical health and well-being			
	Total		Yes		No		Yes		No		Yes		No	
	*%*	*n*	*%*	*n*	*%*	*n*	*%*	*n*	*%*	*n*	*%*	*n*	*%*	*n*
**Talk about the war with parents**	70.4	278	12.9	36	87.1	242	11.9	33	89.1	237	19.1	53	80.9	225
**Do not talk about the war with parents**	29.6	117	8.5	10	91.5	107	6.0	7	94.0	110	8.5	10	91.5	107
			*p=0.213*				*p=0.077*				*p= 0.009*			
			**Emotions and mood**				**Ability to concentrate and learn**				**Hobbies**			
	Total		Yes		No		Yes		No		Yes		No	
	*%*	*n*	*%*	*n*	*%*	*n*	*%*	*n*	*%*	*n*	*%*	*n*	*%*	*n*
**Talk about the war with parents**	70.4	278	55.4	154	44.6	124	15.8	44	84.2	234	9.4	26	90.6	252
**Do not talk about the war with parents**	29.6	117	33.3	39	66.7	78	13.7	16	86.3	101	8.5	10	91.5	107
			*p=0.00006*				*p=0.586*				*p=0.799*			
			**Behaviour**				**Appetite**				**Sleep**			
	Total		Yes		No		Yes		No		Yes		No	
	*%*	*n*	*%*	*n*	*%*	*n*	*%*	*n*	*%*	*n*	*%*	*n*	*%*	
**Talk about the war with parents**	70.4	278	18.3	51	81.5	227	7.2	20	92.8	258	15.8	44	84.2	234
**Do not talk about the war with parents**	29.6	117	14.5	17	85.5	100	3.4	4	96.6	113	9.4	11	90.6	106
			*p=0.359*				*p=0.152*				*p=0.092*			

Lithuanian youth engagement in aid for Ukraine

57.0% of the teenagers responded that they contributed to helping Ukrainians by volunteering and participating in charitable activities. Slightly more than a half (51.4%) of the teenagers who completed the survey noted that they had communicated with Ukrainians who had fled to Lithuania due to the war in Ukraine. However, only one-fifth (20.2%) maintain constant contact and communication with them (Table 4).

**Table 4:** Lithuanian adolescents’ answers about communication with Ukrainians who came to Lithuania

	Yes		No	
	*%*	*n*	*%*	*n*
**Have you ever contributed to helping Ukrainians (volunteering, participating in charity work, etc.)?**	57.0	n=225	43.0	n=170
**Have you had any interaction with Ukrainians who fled to Lithuania because of the war in Ukraine?**	51.4	n=203	48.6	n=192
**Do you maintain regular contact and communication with Ukrainians who fled to Lithuania because of the war and are now living here?**	20.2	n=80	79.8	n=315

It was observed that those adolescents who contributed to helping Ukrainians were statistically significantly more likely to notice that the ongoing war would affect their communication with family members (n=33, 14.7%), (*p*=0.031), emotions and mood (n=126, 56.0%), (*p*=0.001), compared to those who did not contribute (Table 5).

**Table 5:** Comparison between adolescents who contributed and who did not contribute to helping Ukrainians regarding changes in their habits in the context of the ongoing war in Ukraine

			Communication with family members				Communication with friends				Physical health and well-being			
	Total		Yes		No		Yes		No		Yes		No	
	*%*	*n*	*%*	*n*	*%*	*n*	*%*	*n*	*%*	*n*	*%*	*n*	*%*	*n*
**Contributed to helping Ukrainians**	56.9	225	14.7	33	85.3	192	11.1	25	88.9	200	18.2	41	81.8	184
**Do not contributed to helping Ukrainians**	43.1	170	7.6	3	92.4	157	8.8	15	91.2	155	12.9	22	87.1	148
			*p=0.031*				*p=0.456*				*p= 0.156*			
			**Emotions and mood**				**Ability to concentrate and learn**				**Hobbies**			
	Total		Yes		No		Yes		No		Yes		No	
	*%*	*n*	*%*	*n*	*%*	*n*	*%*	*n*	*%*	*n*	*%*	*n*	*%*	*n*
**Contributed to helping Ukrainians**	56.9	225	56.0	126	44.0	99	18.2	41	81.8	184	10.7	24	8.3	201
**Do not contributed to helping Ukrainians**	43.1	170	39.4	67	60.6	103	11.2	19	88.8	151	7.1	12	92.9	158
			*p=0.001*				*p=0.053*				*p=0.217*			
			**Behaviour**				**Appetite**				**Sleep**			
	Total		Yes		No		Yes		No		Yes		No	
	*%*	*n*	*%*	*n*	*%*	*n*	*%*	*n*	*%*	*n*	*%*	*n*	*%*	
**Contributed to helping Ukrainians**	56.9	225	20.4	22	79.6	179	7.1	16	92.9	209	15.1	34	84.9	191
**Do not contributed to helping Ukrainians**	43.1	170	12.9	22	87.1	148	4.7	8	95.3	162	12.4	21	87.6	149
			*p=0.051*				*p=0.322*				*p=0.433*			

Adolescents’ fears about the war in Lithuania

More than a half of the adolescents who filled in the questionnaire feel afraid that the war could also begin in Lithuania, and 40.0% do not feel protected from war (Table 6).

**Table 6:** Adolescents’ fears about the war in Lithuania

	Yes		No		I do not know	
	*%*	*n*	*%*	*n*	*%*	*n*
**Do you feel safe, protected from war?**	24.3	96	40.0	158	35.7	141
**Do you feel afraid that the war could start in Lithuania as well?**	60.8	240	18.7	74	20.5	81

### Open Question

In their answers to the open question, where they were given an opportunity to add to their previous answers, the adolescents mentioned that the war in Ukraine was causing concern, and that it had an impact on their daily lives. Some participants admitted the profound emotional impact that the war in Ukraine had on them, ranging from anxiety and fear to anger and growing patriotism. Whereas, many opined that they had come to value their country and freedom more and had become more sensitive to security issues. The adolescents wrote that they support the difficult struggle of Ukrainians and hope for a quick end to the war.

There are few examples of adolescents’ thoughts: *Lithuania’s NATO membership brings peace, but it’s still unsettling; Ever since the war started, I’ve been thinking about ‘what-ifs’, and it often makes me anxious; I would just like to mention that I think people in Lithuania do not take the war as seriously as they should. This war is happening so close to us that it could all come to our country any day or night; There is a fear that Lithuania will be attacked, but the consequences of such an event on a global scale scare me more; I really hope that the war will end soon, I wish the Ukrainian people strength, I can’t even imagine what they must endure; I think that, by seeing Ukraine fighting for its freedom every day, we can understand the importance of a free homeland; I wish this was talked about more, I volunteer at 3 foundations every day, and seeing people’s ‘fatigue’ from war affects my emotional health even more, because we have no right to be tired of war*.

## Discussion

The war in Ukraine and the atrocities committed by Russia have affected millions of Ukrainians and their supporters around the world [6]. Adolescents are a sensitive group in society. They go through many changes at their age and are in the process of revealing their feelings and forming their worldviews [2]. Adolescents who have experienced traumatic events tend to suppress and keep their feelings and experiences to themselves, which promotes the development of a depressed mood [9]. The ongoing war inflicts damage on mental health, and many of its related consequences extend beyond the points of conflict [10]. There is little research on how the war in Ukraine is affecting adolescents in neighbouring countries; hence, this research is manifested by novelty and relevance. Although teenagers living in Lithuania have not yet faced direct war atrocities, they are nevertheless exposed to information about the war in Ukraine on the media, which can have a strong impact on their emotional well-being. Children and adolescents indirectly affected by war do not have to immediately experience the drastic consequences of war: separation from family or loved ones, abandonment of homes and hometowns, loss of friends, but they experience social threat when participating on a collective level, hearing news about ongoing hostilities, or interacting with refugees who have become victims of war. A war in a neighbouring country can create a sense of danger and vulnerability, which is also the essence of the trauma experience [2]. This study expands on the emotional impact of the ongoing war in Ukraine on Lithuanian adolescents. While their daily routines, school, hobbies, and sleep remain unaffected, nearly a half of the respondents reported changes in their mood due to war events. A significant portion also expressed fear that the conflict could spread to Lithuania, reflecting heightened insecurity among youth in geopolitically sensitive regions. Previous studies have shown that adolescents are particularly vulnerable to stressors caused by geopolitical conflicts. Research on war-related anxiety in youth suggests that media exposure plays a key role in amplifying fear and uncertainty [11]. Similarly, our findings indicate that those who frequently engage with war-related news or discuss the war with their parents are more likely to report emotional distress. This aligns with research that found that higher media consumption during crises can increase psychological strain, especially in young people [5]. However, selective engagement with war-related content may also serve as a coping mechanism, as seen in studies on emotional distancing during prolonged crises [13]. Notably, girls were more likely to experience emotional effects from the war, which is a trend observed in other research on trauma and stress response differences between genders [14]. This could be due to greater emotional sensitivity and empathy, as well as increased engagement in discussions about war-related events.

The issues surrounding children’s mental health are complex, and they include not only health but also the social environment [15]. Most teenagers seek assistance of mental health centres due to increased anxiety levels. Doctors, psychologists, and other health professionals often purposefully avoid to ask how the war in Ukraine affects teenagers, and how they react to environmental changes. Increased anxiety, fears, sleep, appetite problems, changes in mood and behaviour problems might be reaction to the war in Ukraine.

The opportunity to help those affected by the war and contribute to the best possible outcome of the events helps children and adolescents feel better. Lithuanians strongly support the Ukrainians fighting for the freedom of their country. A study conducted in Romania found that adolescents who volunteered and contributed to helping war-affected Ukrainians reported greater moral uplift, but also greater peritraumatic dissociative experiences [2]. Whereas, results of our study have revealed that teenagers who contributed to helping Ukrainians were statistically significantly more likely to notice changes in emotions and mood against the backdrop of the ongoing war in Ukraine (*p*=0.001).

### Implications and future directions

Given these findings, it is essential to create safe spaces where adolescents can express their concerns and process their emotions. Studies have shown that open discussions in schools can reduce war-related anxiety and promote emotional resilience [13]. Moreover, media literacy programs could help young people critically engage with war-related news, while reducing the risk of misinformation and unnecessary distress [14].

Future research could explore long-term psychological effects of living under the perceived threat of war. Similar studies on children in conflict-adjacent countries have found that persistent exposure to crisis narratives can shape worldviews, trust in institutions, and mental well-being into adulthood [1]. Understanding these long-term consequences could help policymakers and educators develop targeted support systems for adolescents growing up in times of geopolitical instability.

### Limitations of the study

This study has several limitations. First of all, the study sample was drawn from only major cities of Lithuania. Consequently, the findings may be not fully generalizable to young people from smaller towns or rural areas. In the future, the sample could be expanded to include schools in smaller towns and villages, and to investigate whether there are differences in opinions and attitudes across different geographical and socio-cultural contexts. Second, participation in the study was voluntary, and the survey was conducted anonymously. Therefore, the results reflect only the views of those adolescents who agreed to participate in the study. The opinions of individuals who did not agree to participate in the study and did not fill out the questionnaire remain unknown. It is unclear how many potential respondents chose not to complete the questionnaire. This self-selection bias may have influenced the findings. Finally, the study included only adolescents aged 16 years and older. As attitudes and perceptions may differ across developmental stages, future studies could also explore the views of younger adolescents so that to reflect the perspectives of younger adolescents.

## Conclusions

The ongoing war in a neighbouring country, constant media coverage, and the looming threat of conflict impact the mental well-being of the Lithuanian youth, particularly their emotions and mood. Although Lithuanian adolescents are not being directly affected by the war, its psychological impact is evident. Many adolescents experience emotional distress and fear about the potential expansion of the conflict. This highlights the importance of researching and monitoring the emotional health of children and adolescents, even in peaceful regions. Encouraging open dialogue, providing mental health resources, and promoting critical media consumption is crucial in helping young people navigate these uncertain times. By addressing their concerns in a structured way, we can foster resilience and ensure that adolescents are better equipped to process global crises in a healthy manner.
